# Myocardial Deformation and Its Relation to Ventricular Shape, Preload, and Afterload in Fetuses with Suspected Coarctation of the Aorta

**DOI:** 10.1007/s00246-025-03878-6

**Published:** 2025-05-08

**Authors:** Trisha V. Vigneswaran, Tomas Woodgate, Joao Rato, Reza Razavi, John M. Simpson

**Affiliations:** 1 School of Biomedical Engineering & Imaging Sciences, King’s College London, London, SE1 7EH UK; 2 Department of Congenital Heart Disease, Evelina London Children’s Hospital, Guy’s & St Thomas’ NHS Trust, London, SE1 7EH UK; 3 Department of Pediatric Cardiology, Hospital Santa Cruz-Centro Hospitalar Lisboa Ocidental, Lisbon, Portugal

**Keywords:** Congenital heart disease, Fetal echocardiogram, Coarctation of the aorta, Myocardial deformation

## Abstract

Detailed characterization of myocardial deformation, ventricular shape, outflow tract size, inflow Doppler patterns, cerebroplacental circulation, and cardiac output of fetuses with suspected coarctation of the aorta (COA) and a control group to gain further insights into differences between these groups. Expectant women were prospectively recruited for assessment during the third trimester of pregnancy and a comparison of echocardiographic characteristics and fetoplacental circulation according to postnatal diagnosis of either confirmed COA (c-COA), false-positive COA (fp-COA), and a control population. There were 42 fetuses recruited with suspected COA of whom 20/42 (48%) had c-COA. Fetuses with c-COA demonstrated lower (less negative) LV global longitudinal strain (LV-GLS) compared to controls (− 20.2% ± 4.3 vs. − 23.1% ± 2.7, *p* = 0.01) and a non-significant trend to lower strain in the fp-COA group (LV-GLS: − 20.7% ± 5.0, *p* = 0.053) compared to controls. RV-GLS was significantly reduced in the fp-COA group compared to the c-COA and control groups (fp-COA: − 19.8% ± 4.5, c-COA: − 23.1% ± 4.4, control: − 23.5% ± 3.6, *p* = 0.04). C-COA and fp-COA had a less spherical (narrower) LV, shorter LV, and a more spherical RV compared to controls. The arterial duct diameter was larger in c-COA compared to fp-COA and controls. When analyzed according to diagnostic group, determinants of GLS and sphericity index differed between groups. For c-COA, there was correlation between LV-GLS and RV-GLS (r = − 0.51, *p* = 0.021). Determinants of LV-GLS in fp-COA were distal transverse aortic arch z-score and umbilical artery PI (*p* = 0.026, *p* = 0.037 respectively). The only determinant of RV-GLS in the FP-COA was arterial duct z-score (*r* = − 0.51, *p* = 0.019). There are measurable differences in functional parameters between c-COA, fp-COA, and controls. The hemodynamic characteristics of c-COA and fp-COA merit further study and should include study of the RV and cerebroplacental evaluation.

**Supplementary Information:**

The online version contains supplementary material available at 10.1007/s00246-025-03878-6.

## Introduction

Coarctation of the aorta (COA) is suspected prenatally when there is asymmetry in the size of the great arteries and/or ventricles with dominance of the right-sided cardiac structures and a variable degree of hypoplasia of the left ventricle (LV) and aortic arch. During fetal life, due to patency of the arterial duct, critical obstruction of the aortic arch does not occur. In practice, newborn infants with prenatal suspicion of COA typically undergo sequential echocardiographic assessment to check for development of obstruction to the aortic arch as the arterial duct closes. In many cases, COA does not develop following ductal closure [1–3]. The etiology of such false-positive cases (fp-COA) remains obscure, and the current approach is resource intensive as well as the diagnostic doubt causing anxiety for expectant parents. Gestation-adjusted dimensions of the aortic arch during fetal life might be expected to predict COA, but there is no threshold dimension with good sensitivity and specificity [3, 4], and this is also the case for fetal cardiac MRI metrics [5]. Most studies have focused on the size of left-sided cardiac structures and/or the shape of the aortic arch to predict postnatal development of CoA. There have been few studies of fetal circulatory hemodynamics and cardiac function in the setting of coarctation of the aorta in utero*,* and these have focused on parameters derived from ventricular inflow, cardiac output, and ventricular shape*.*[6, 7] Myocardial deformation, by speckle-tracking analysis, has been reported in three studies. Pilot data from our own unit demonstrated less myocardial deformation of the LV in c-COA compared to control fetuses [8], a second study comparing c-COA and fp-COA did not demonstrate any difference between the two groups in LV or RV strain [9] and more recently, a retrospective study also demonstrated less LV myocardial deformation in c-COA compared to fp-COA. [10]

The aim of this study was to characterize myocardial deformation, ventricular shape, inflow Doppler patterns, cerebroplacental circulation, and cardiac output of fetuses with suspected COA and a control group to gain further insights into differences between these groups.

First, we hypothesized that myocardial deformation, ventricular shape, ventricular output, ventricular inflow Dopplers, and indices of afterload would be different in fp-COA, c-COA, and control fetuses. Second, it was hypothesized that myocardial deformation is related to ventricular shape, ventricular inflow Dopplers, and indices of afterload in fetuses suspected to have COA.

## Methods

### Study Design

Our study group was taken from the Intelligent Fetal Imaging and Diagnostics-2 (iFIND2) research study (REC: 07/H0707/105, REC 14/LO/1806). Women who were aged 18 years or older were recruited. The overall study design included fetal MRI and so women were excluded if they weighed more than 125 kg or if there was a contraindication to MRI. Written consent was obtained for participation. The IFIND-2 protocol included a third trimester (≥ 28 weeks’ gestation) ultrasound assessment in a clinical research facility under a standardized research protocol including advanced fetal and placental imaging. Participants were unable to adjust positioning during the time-limited (40 min) scan due to the placement of ultrasound probe tracking markers over the maternal abdomen. Expectant women were scanned supine with head-up at 30 degrees, and the research scan was terminated if adjustments to position were required.

### Patients

Inclusion criteria for the COA study were prenatal suspicion of COA as diagnosed by a consultant fetal cardiologist at either Evelina London Children’s Hospital or King’s College Hospital at the mid-gestation scan according to our previously described approach [4]. COA was suspected when there was ventricular and/or arterial disproportion, generally when there was major discrepancy of the size of aortic and ductal arches or the distal transverse aortic arch Z score was more negative than − 2 [4]. Volunteers with uncomplicated pregnancies were also recruited as controls. Exclusion criteria for the deformation study were aortic stenosis (pulsed wave Doppler velocity > 99 th centile [11]) and maternal diabetes mellitus. Fetal ultrasonography took place between June 2016 and January 2019.

The subjects were classified into three groups: confirmed COA (c-COA), false positive for COA (fp-COA), and a normal control population. C-COA was defined as the development of COA in infancy requiring surgical COA repair. This final decision for surgical repair of COA was made after birth and independent of our research findings at our institutional (Evelina London Children’s Hospital) multidisciplinary surgical meeting which includes specialists in paediatric cardiology, congenital cardiac surgery, pediatric intensive care, and neonatology. All patients referred for surgical coarctation repair had the diagnosis confirmed at the time of surgery.

The sample size was calculated for a study with 90% power and *p* = 0.05 to detect a difference of 6.6% in LV global strain as per a retrospective pilot study assessing myocardial deformation undertaken in our department [8]. This determined a sample size of 11 patients per group. Allowing for 50% of drop-out or unfeasibility of image analysis, we determined, at least, a sample of 17 patients for each group.

### Ultrasound Assessment

**Fig. 1:**
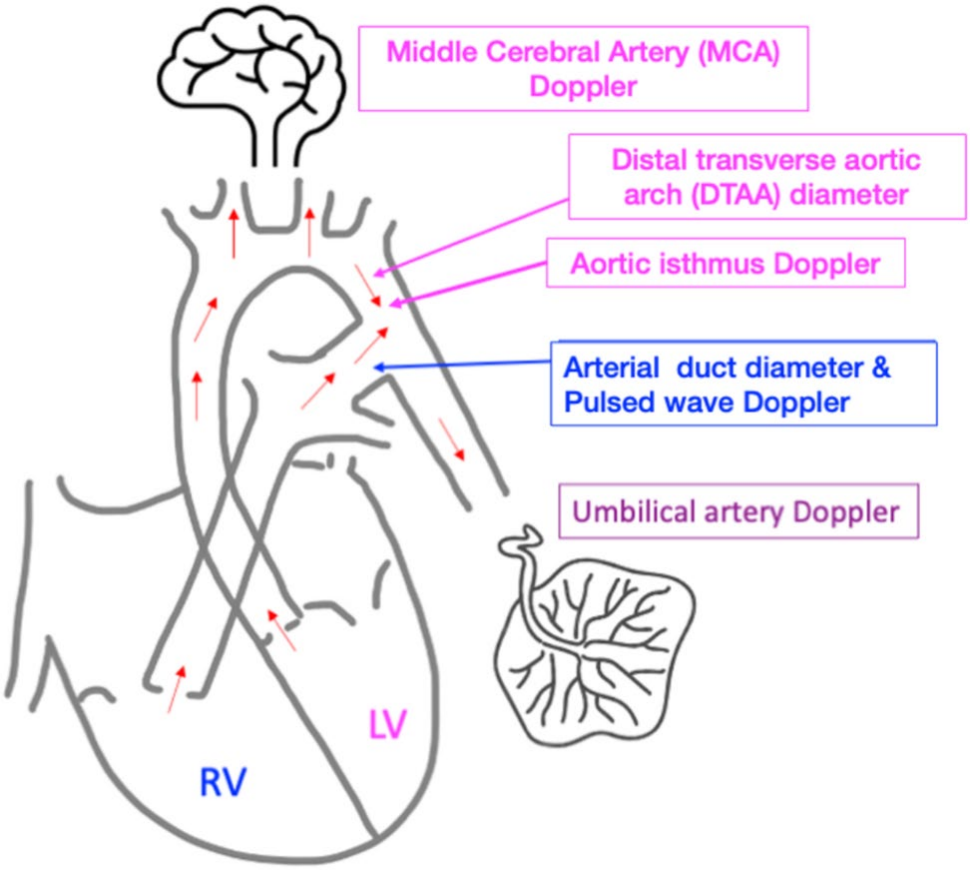
Graphical of the fetal circulation demonstrating potential regions which may influence myocardial deformation

The physiology of the prenatal circulation differs from postnatally (Fig. 1) with a parallel rather than series arrangement. The left ventricle faces the resistance of flow into the aortic arch, cerebral, and placental circulation whereas the RV faces the resistance of the arterial duct, pulmonary vascular resistance, and placental vascular resistance. Middle cerebral artery (MCA) and umbilical artery (UA) pulsatility index (PI) were used to assess cerebral and placental vascular resistance, respectively. The size of vessels, e.g., distal transverse aortic arch (DTAA) and arterial duct were also measured as resistance will reflect their size amongst other variables. The ultrasound assessment was designed to measured size of ventricles, outflow tracts, cardiac output, myocardial deformation, and downstream vascular resistance.

Echocardiograms were performed by a fetal cardiologist. Acquisitions of the four-chamber view, atrioventricular valve diameters, right and left ventricular lengths (RVL, LVL) from the midpoint of the valve annulus to the myocardium of the ventricular apex, and the midpoint width of the ventricles (RVW, LVW) were measured and z-scores calculated according to Krishnan et al. [12]. Outflow tract diameters, inflow Dopplers, and outflow tract Dopplers were measured, and z-scored according to published reference ranges from our institution [11, 13]. Sphericity index (SI) was calculated using the formula: LVW/LVL [14]. Right and left ventricular cardiac output (RV-CO, LV-CO) were measured using the standard pulsed wave Doppler-valve area method (0.785 × *D*^2^ x VTI x FHR), where D = diameter of the pulmonary/aortic valve, FHR = fetal heart rate and VTI was calculated using pulsed wave Doppler with the cursor place on the pulmonary/aortic valve and indexed to estimated fetal weight (EFW) calculated by the Hadlock formula using femur length, abdominal circumference, and biparietal diameter [15] where feasible pulsed wave Doppler in the arterial duct and DTAA was obtained in cases of suspected COA. Middle cerebral artery (MCA) and umbilical artery (UA) Doppler were ascertained and z-scores calculated [16]. Structural cardiac measurements were made prospectively during the live scan. Indices which may reflect ventricular afterload were reported in relation to GLS and SI (Fig. 1*, *Table 1).

**Table 1:** Assessment of variables relating to ventricular preload and afterload

	Ventricular inflow pattern	Vascular measurements
Right ventricle	Tricuspid valve E, A peak velocity	Arterial duct size Arterial duct PI/VTI Arterial duct VTI Umbilical artery PI
Left ventricle	Mitral valve E, A peak velocity	Distal transverse aortic arch size Distal transverse aortic arch VTI/PI Middle cerebral artery PI Umbilical artery PI

Abbreviations *PI* pulsatility index, *VTI* velocity time integral

Ultrasounds were performed using the Philips EPIQ system using a C9-2, C5-1 curvilinear probes or X6 probe, and measurements were undertaken on Qlab10.3 and Q station platforms (Philips Medical Systems, Zoetermeer, The Netherlands).

### Myocardial Deformation

**Fig. 2:**
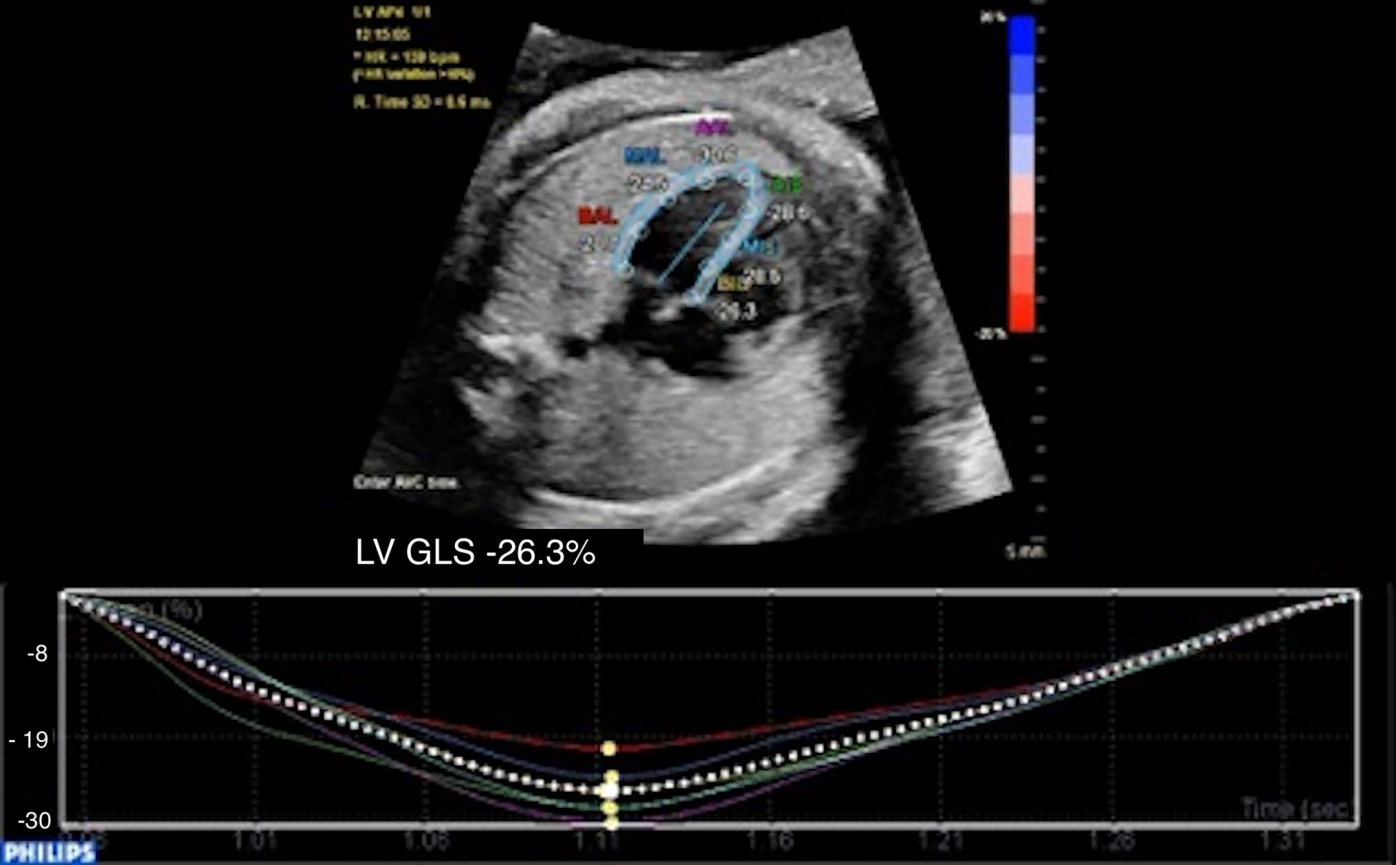
Example of LV deformation analysis in a fetus

Zoomed four-chamber acquisitions without acoustic shadow were acquired specifically for speckle-tracking assessment with the highest achievable frame rate by optimization of depth and sector width. DICOM loops were acquired during periods of fetal inactivity aiming for a minimum frame rate of 60 frames per second [17] and stored at native frame rate for speckle-tracking assessment. One single cardiac cycle was chosen by selecting two consecutive end-diastolic frames (mitral valve closure). The cardiac cycle was chosen for analysis based on good endocardial border definition and the absence of fetal movement/respiratory motion. Analysis of myocardial deformation was undertaken prior to the birth of the infant without knowledge of the postnatal outcome of the baby. The right and left ventricles (RV/LV) were analyzed separately. Markers were placed on the basal septum, basal free wall, and the apex of the ventricle in the frame before closure of the mitral/tricuspid valve in the 4-chamber view and an automated tracking of the myocardium activated (Fig. 2). For the RV, only the free wall segments were analyzed (RVFW-GLS) as the segments of the interventicular septum were analyzed as part of the LV. Quality of tracking was assessed subjectively by observation of the tracking of the six segments throughout the cardiac cycle. 2D global longitudinal strain (GLS) was automatically generated. Measurements were repeated on a separate cardiac cycle (inter-cycle variability) and on the same cardiac cycle for intra-user variability. Good inter-user variability has previously been demonstrated by our group [18]. Myocardial deformation analyses were performed using the aCMQ software (QLAB 10.3; Philips Medical System, Zoetermeer, The Netherlands).

### Statistical Analysis

Data are described as mean ± standard deviation or median (interquartile range) for non-Gaussian data. Group comparisons with either one-way ANOVA with post hoc testing or Kruskal–Wallis test according to normality and correlation was assessed using Pearson’s test. Multiple linear regression was used to assess determinants of myocardial deformation, ventricular shape, and cardiac output. Intra-observer correlation coefficient was used to assess intra-user variability (20% cases reanalysed) for deformation analysis. Analysis was undertaken on Prism V7 (Graphpad software Inc. CA, USA), SPSS V26.0 for MAC (IBM Corp. Chicago, IL, USA), and STATA version 17 (StataCorp LLC).

## Results

There were 42 fetuses recruited into this study with suspected COA of whom 20/42 (48%) were confirmed to have COA in infancy (all of whom were diagnosed and operated in the neonatal period). There was no significant difference in baseline demographics of the c-COA, fp-COA, and control groups (Table 2). The LV and MV size was significantly smaller in the fp-COA and c-COA cases compared to controls (Table 3). LV-GLS was significantly reduced (less negative) in the fetuses with c-COA (*p* = 0.01) and approached a significant reduction in the fp-COA group (*p* = 0.053) compared to controls. The RVFW-GLS was significantly reduced in the fp-COA group compared to the c-COA and control groups (Table 3).

**Table 2:** Background demographics of the cohort

	Coarctation *n* = 20	False positive *n* = 22	Controls *n* = 38	*p *value
Median gestation, weeks (IQR)	30.5 (30–32)	31 (31–32)	31 (30–31)	0.069
Median maternal age, years (IQR)	31 (27–34)	31 (30–36)	32 (29–36)	0.568
Maternal weight, kg	78.4 ± 10.9	77.4 ± 10.5	75.9 ± 12.3	0.722
Maternal BMI, kg/m^2^	27.5 ± 3.5	29.1 ± 3.8	28.1 ± 5.1	0.494
Maternal hypertension	0	1/23	0	0.18
Birth weight, kg	3.0 ± 0.67	2.96 ± 0.37	3.34 ± 0.54	0.779*
Birth gestation, weeks	38 (37–38)	38 (38–39)	40 (39–41)	0.475*

Abbreviations: *IQR* interquartile range, *BMI* body mass index. *represents p value comparison of c-COA and fp-COA

**Table 3:** Fetal cardiac size, shape, myocardial deformation assessments

Variable	Confirmed-COA *n* = 20	False-positive COA *n* = 22	Controls *n* = 38		*p* value		
				ANOVA	c-COA vs. fp-COA	c-COA vs. control	Fp-COA vs. control
**Cardiac size**							
Mitral valve z-score	− 1.8 ± 1.6^†^	− 1.1 ± 1.3^§^	− 0.02 ± 1.1^†§^	< 0.001	0.30	< 0.001	0.007
LV width, mm	8.4 ± 1.7^†^	9.8 ± 2.7^§^	11.4 ± 1.9^†§^	< 0.001	0.09	< 0.001	0.09
LV length, mm	21.0 ± 3.4^†^	22.4 ± 3.6	23.3 ± 4.4^†^	0.13	0.42	0.01	0.30
LV length z-score	− 2.8 ± 1.3^†^	− 2.6 ± 1.2	− 1.8 ± 1.3^†^	0.01	0.89	0.03	0.06
Tricuspid valve z-score	0.5 ± 1.4	0.4 ± 1.7	− 0.2 ± 1.0	0.11	0.99	0.16	0.30
RV width, mm	13.9 ± 3.1	13.3 ± 2.9	12.4 ± 2.4	0.12	0.94	0.25	0.43
RV length, mm	21.6 ± 3.1	21.9 ± 5.7	23.5 ± 3.2	0.17	0.58	0.08	0.67
RV length z-score	− 1.7 ± 1.1	− 1.6 ± 1.2	− 1.1 ± 1.1	0.1	0.95	0.123	0.26
**Cardiac shape**							
LV sphericity index	0.41 ± 0.11^†^	0.45 ± 0.14	0.51 ± 0.16^†^	0.03	0.52	0.02	0.31
RV sphericity index	0.61 ± 0.12^†^	0.60 ± 0.15	0.53 ± 0.11^†^	0.03	0.67	0.04	0.27
**Myocardial deformation analysis**							
LV-GLS, %	− 20.2 ± 4.3^†^	−20.7 ± 5.0	− 23.1 ± 2.7^†^	0.01	0.95	0.03	0.10
RV free wall GLS, %	− 23.1 ± 4.4^★^	− 19.8 ± 4.5^★§^	− 23.5 ± 3.6^§^	0.04	0.056	0.93	0.007
Heart rate, bpm	140 ± 11	135 ± 15	141 ± 11	0.34	0.55	0.98	0.40
Frame rate, fps	66 ± 23	78 ± 17	76 ± 24	0.15	0.20	1.0	0.27
**Morphology**							
Bicuspid aortic valve	12/20	8/22	0	0.13	–	–	–

^†^significant difference on post hoc testing between confirmed coarctation and control group

^§^significant difference on post hoc testing between false-positive and control groups

^★^significant difference on post hoc testing between confirmed coarctation and false-positive groups

ANOVA and post-hoc analysis p values are provided

**Fig. 3:**
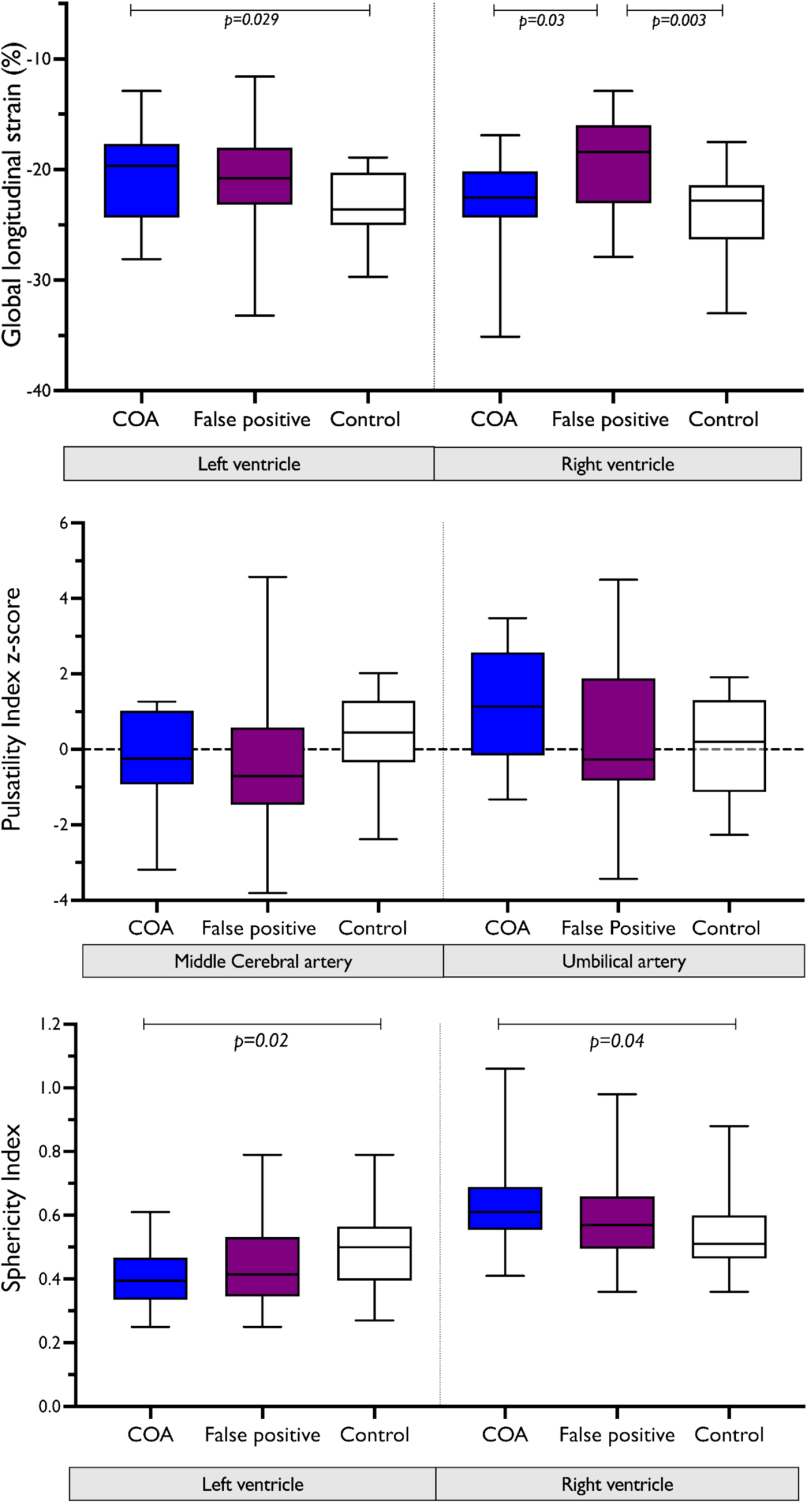
Box-whisker plot comparison of (**a**) global longitudinal strain. **b** Cerebral and placental resistance. **c** sphericity index

With regard to the shape of the ventricles, the fetuses with c-COA had a less spherical (narrower) LV, shorter LV, and a more spherical RV compared to controls (Table 3). The c-COA did not differ in ventricular shape compared to fp-COA cases. Mitral and tricuspid inflow patterns did not differ between the 3 groups (Table 4). Regarding vascular variables, DTAA z-score was lower in the c-COA and fp-COA cases compared to the control group. MCA-PI and umbilical artery PI z-scores were not significantly different between c-COA, fp-COA, and control groups (Fig. 3b). The arterial duct was significantly larger in the c-COA group compared to fp-COA and control groups. Comparisons of all ventricular size, inflow Doppler patterns, and vascular metrics are shown in Table 3.

**Table 4:** Vascular and hemodynamic measures

Variable	Confirmed COA *n* = 20	False-positive COA *n* = 22	Controls *n* = 38	*p value*
**Determinants of preload**				
MV E	45.8 ± 9.4	40.6 ± 7.6	42.8 ± 16.6	*0.26*
MV E z-score	2.0 ± 1.7	1.1 ± 1.3	1.5 ± 2.8	*0.25*
MV A	57.3 ± 12.4	52.5 ± 10.9	51.1 ± 6.4	*0.34*
MV A z-score	0.3 ± 0.9	0.0 ± 0.8	− 0.1 ± 0.4	*0.33*
MV inflow VTI	7.21 ± 1.3	6.67 ± 1.61	5.97 ± 0.54	*0.15*
TV E	41.3 ± 12.2	45.5 ± 10.2	41.3 ± 5.9	*0.45*
TV E z-score	0.5 ± 1.5	0.9 ± 1.3	0.5 ± 0.6	*0.60*
TV A	55.0 ± 13.7	54.3 ± 13.1	55.4 ± 8.5	*0.98*
TV A z-score	0.1 ± 0.1	0.1 ± 0.1	0.1 ± 0.0	*0.76*
TV inflow VTI	6.68 ± 1.5	7.07 ± 2.14	6.19 ± 1.0	*0.54*
**Vascular metrics**				
Distal transverse aortic arch (mm)	2.6 ± 0.7^†^	2.9 ± 0.8^§^	3.6 ± 0.8^†§^	< *0.001*
Distal transverse aortic arch z-score	− 3.1 ± 1.5^†^	− 2.6 ± 1.7^§^	− 0.7 ± 1.8^†§^	< *0.001*
Arterial duct (mm)	5.0 ± 0.9^†^	4.4 ± 1.1	4.2 ± 0.8^†^	*0.006*
Arterial duct z-score	2.5 ± 2.1^†★^	1.0 ± 2.4^★^	0.6 ± 1.9^†^	*0.004*
MCA-PI z-score	− 0.133 ± 1.18	− 0.47 ± 1.81	0.29 ± 1.29	*0.31*
Umbilical Artery PI z-score	1.14 (− 0.16–2.57)	− 0.27 (− 0.83–1.88)	0.15 (− 1.13–1.31)	*0.21*
Cerebroplacental ratio z-score	− 1.02 ± 1.34	− 0.79 ± 2.19	− 0.09 ± 1.15	*0.25*
**Hemodynamic parameters**				
Aortic isthmus PI	3.01 ± 1.39	3.03 ± 0.84	–	*0.95*
Aortic isthmus VTI (forward)	14.65 ± 4.01	12.41 ± 3.11	–	*0.10*
No. with reverse flow in aortic isthmus	1/13	6/16	–	*0.15*
Arterial duct PI	2.61 (2.27–2.88)	2.3 (2.13–2.45)	–	*0.08*
Arterial duct VTI	14.76 ± 3.64	15.37 ± 5.62		*0.76*
Aortic isthmus: arterial duct VTI	1.11 ± 0.42	0.94 ± 0.46	–	*0.35*
LV cardiac output, ml/kg/min	96 (68–137)^†^	104 (90–133)^§^	135 (103–191)^†§^	*0.048*
RV cardiac output, ml/kg/min	335 (197–417)	263 (223–374)	338 (265–398)	*0.61*
Combined cardiac output, ml/kg/min	441 (265–569)	377 (322–500)	501 (402–548)	*0.20*
Ratio of LV:RV cardiac output, ml/kg/min	0.33 (0.25–0.41)^†^	0.38 (0.25–0.46)	0.45 (0.35–0.68)^†^	*0.11*

(*p*<0.05)

^†^significant difference on post hoc testing between coarctation and control group (*P*<0.05)

^§^significant difference on post hoc testing between false-positive and control groups(*P*<0.05)

^★^significant difference on post hoc testing between coarctation and false-positive groups (*P*<0.05)

Abbreviations *VTI *velocity time integral, *MCA* middle cerebral artery, *UA* umbilical artery, *PI* pulsatility index

**Fig. 4:**
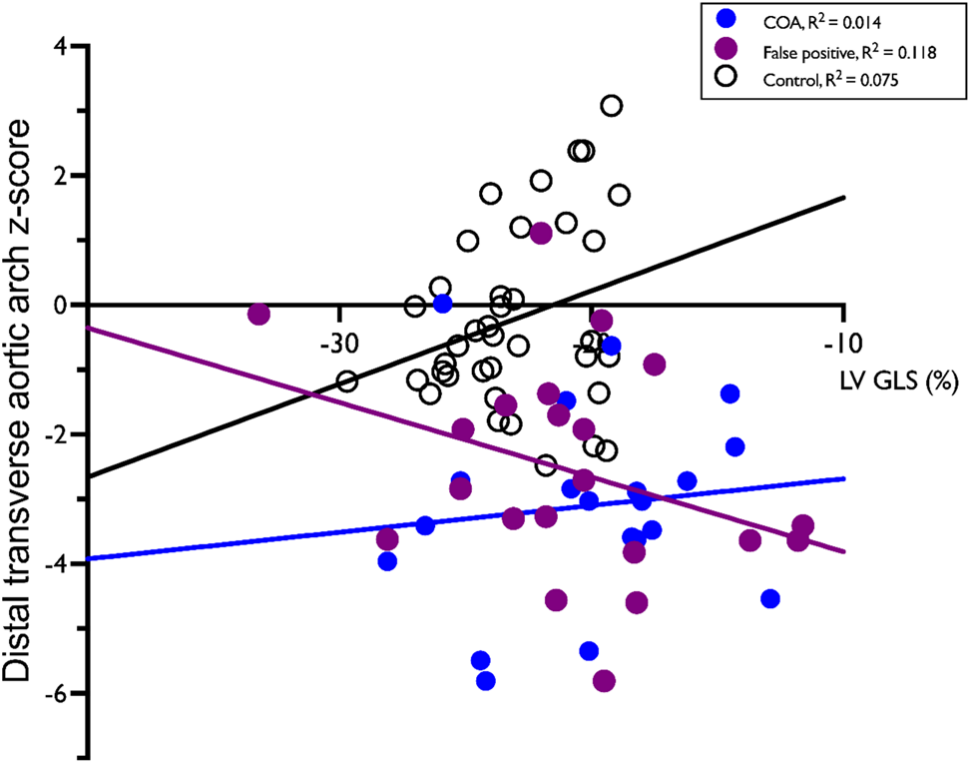
Correlation of LV-GLS to aortic arch size

When analyzed as the whole cohort, there was negative correlation between LV-GLS and DTAA z-score (*r*^2^ = 0.05, *p* = 0.046) (Fig. 4). When all cases (fp-CoA, c-CoA, and controls) are included, there was no correlation between LV-GLS or RVFW-GLS or LV-SI or RV-SI with gestational age, frame rate, arterial duct z-score, LV-CO, RV-CO, MCA-PI z-score, UA-PI z-score, CPR, or birthweight. When assessed according to diagnostic group (c-COA, fp-COA, controls) separately, the following significant trends were observed.

**Fig. 5:**
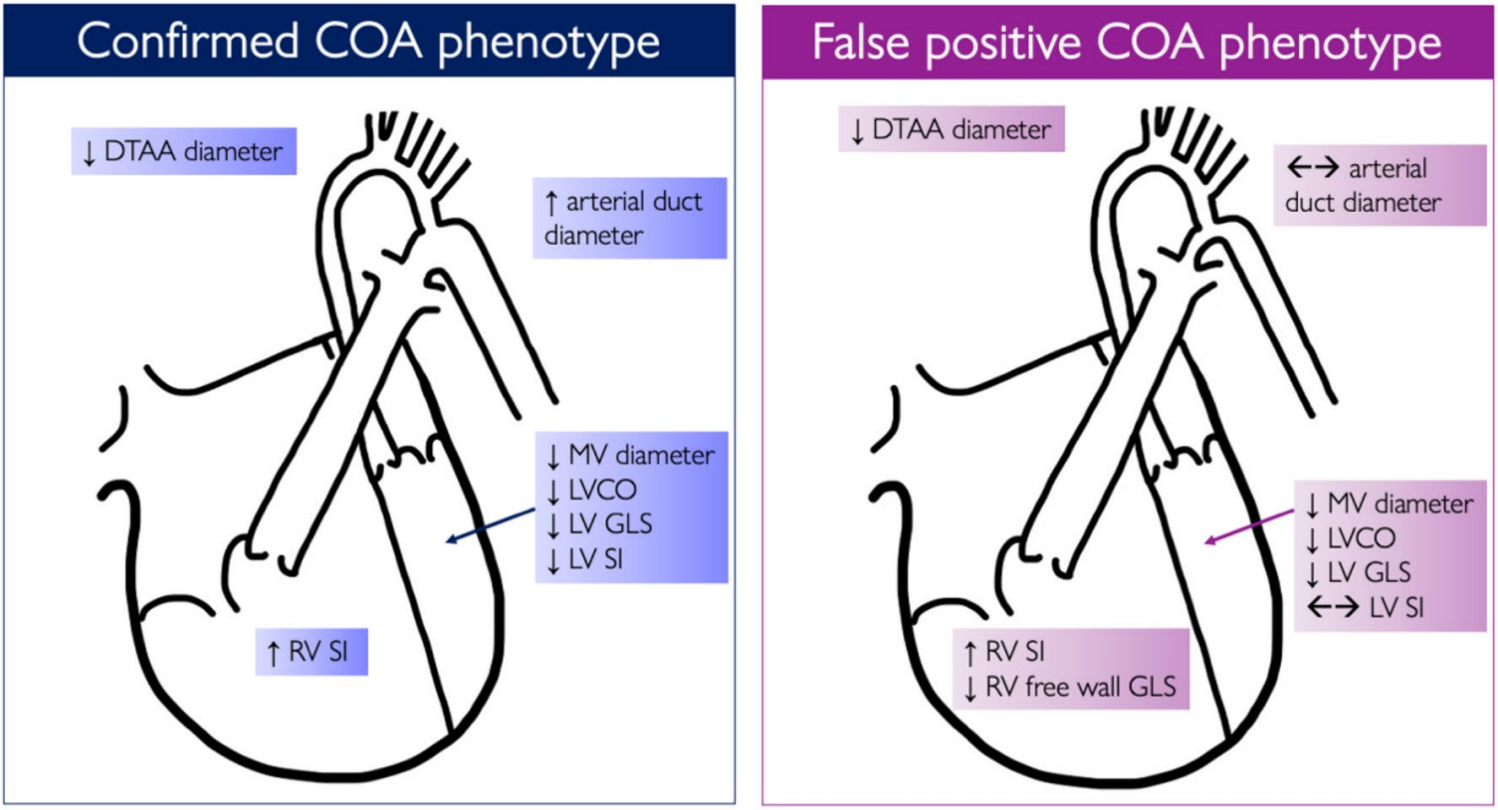
Graphic showing the differences in the phenotype of the c-COA and fp-COA compared to control fetuses

- i) Control group: A negative correlation between LV-GLS and LV-CO (*r* = 0.55, *p* = 0.032) was present and the correlation of LV-GLS and MCA-PI z-score approached significance (*r* = 0.48, *p* = 0.051). Normal fetuses demonstrated a correlation between the shape (sphericity) of the right and left ventricles (*r* = 0.37, *p* = 0.024). Multiple regression equations are provided in Table 5.
- ii) Confirmed-COA group: These fetuses demonstrated less LV deformation, lower LV-CO, a narrower LV, and smaller DTAA z-score compared to control fetuses. A positive correlation was present in c-COA fetuses between LV-GLS and RVFW-GLS (*r* = 0.51, *p* = 0.021). With regard to the RV in c-COA cases, RV-SI positively correlated with UA-PI z-score (*r* = 0.49, *p* = 0.035) and negatively correlated with arterial duct PI. RVFW-GLS was similar to normal fetuses. (Fig. 5).
- iii) False-positive COA group: Regarding vascular markers, the aortic arch size was significantly smaller than normal (z-score: − 2.6±1.7). There was a non-significant correlation of LV-GLS with UA-PI z-score (*r*^2^ = −0.43, *p*=0.057). RVFW-GLS was reduced in the fp-COA group compared to control and c-COA (*p*=0.04). RVFW-GLS demonstrated a negative correlation with arterial duct z-score (*r*^2^ = − 0.261, *p*=0.02). (Figure 5, Supplementary Figure 1)

**Table 5:** Multivariable regression analysis demonstrating associations of myocardial deformation and sphericity index according to underlying diagnostic phenotype

		*R*^2^	Variables in equation	*ß* Coefficient	*p* value
Control	LV-GLS	0.307	Constant LV-CO	− 18.585 − 0.033	< 0.001 0.032
	LV-SI	0.137	Constant RV-SI	0.401 0.259	0.001 0.024
	RV-SI	0.137	Constant LV-SI	0.230 0.528	0.068 0.024
Confirmed COA	LV-GLS	0.261	Constant RV-GLS	− 8.489 0.507	0.089 0.021
	RV-GLS	0.261	Constant LV-GLS	− 12.676 0.516	0.008 0.021
	RV-SI	0.237	Constant umbilical artery PI z-score	0.592 0.050	< 0.001 0.035
False-positive COA	LV-GLS	0.398	Constant DTAA z-score umbilical artery PI z-score	− 23.545 − 1.372 − 1.0	< 0.001 0.026 0.037
	RV-GLS	0.256	Constant arterial duct z-score	− 18.939 − 0.942	< 0.001 0.019

Intra-user variability for deformation analysis was good–excellent when the same cardiac cycle was assessed (LV: 0.95, RV: 0.85) and moderate–good when a different cardiac cycle was assessed (LV: 0.77, RV: 0.72)

## Discussion

### Main Findings of the Study

This study prospectively assessed myocardial deformation, ventricular shape, CO, ventricular inflows, vascular measurements, and resistance in vascular beds in a recruited and consented research cohort of third trimester fetuses in which COA was suspected at the second trimester. Our study differs from the three other studies [6, 9, 19] of myocardial deformation in suspected COA in that ours was a prospective study, the postnatal outcome at the time of measurement was not known, and we included a comprehensive assessment of the size of the aortic arch, arterial duct and resistances in downstream vascular beds. This study is important for those practizing in fetal medicine and fetal cardiology as there is a genuine diagnostic difficulty which is faced in advance of delivery when cardiac asymmetry is identified regarding whether there will be COA or not. Cardiac asymmetry may reflect COA or differences in loading conditions which then causes the cardiac asymmetry and changes in ventricular shape and arterial proportions.

### Phenotype of Fetus with c-COA

Our study confirms that there are both similarities and differences between the fetus with c-COA and fp-COA. As previously reported, the phenotype of fetuses with c-COA is of smaller left heart structures (MV annulus, LV width, DTAA). [3, 4] LV-CO was lower in the c-COA compared to controls and as a possible compensatory factor the arterial duct was larger than normal and the RV has increased sphericity (Fig. 3c). The latter results in maintenance of CCO and is influenced by umbilical artery PI (Table 5). The Doppler velocities and flow across the MV and TV were no different in c-COA compared to controls. There was no significant difference in the cerebrovascular or placental resistances in the c-COA compared to controls. The LV was less spherical in the c-COA, and with respect to myocardial deformation, we have reconfirmed the pattern described previously [8, 9] with lower (less negative) LV deformation in c-CoA compared to controls. Thus, it could be inferred that fetuses with c-COA have a phenotype of hypoplasia of the left heart structures together with decreased LV deformation. Myocardial deformation is affected by preload and afterload [20], but no correlation was observed between LV-GLS and variables related to ventricular afterload (MCA-PI, UA-PI, DTAA z-score, arterial duct size, Table 5). This would be consistent with LV myocardial dysfunction and/or reduced preload which may itself be linked to hypoplasia of the left heart. Primary myocardial dysfunction has been described in adults with repaired COA [21, 22], and therefore, it is plausible that this might be evident from fetal life. Other factors which might impact LV deformation include an alteration in preload by means of reduced right to left shunting or reversed (left to right) flow across the atrial septum which has been described in fetuses with ‘severe’ COA [23]. However, it is not possible to measure flow across the foramen ovale using echocardiography and further assessment with fetal cardiac magnetic resonance imaging may prove insightful in answering this question, but reduced foramen ovale flow was not supported by differences in flow across the mitral or tricuspid valves. Increased arterial stiffness of the pre-coarctation site arterial vasculature is present in both children and adults [24], and this might also influence LV-GLS, but there are no validated means of assessing arterial stiffness in fetuses at present.

### Phenotype of fp-COA

The fp-COA group has similarities and differences to both the c-COA and control groups indicating that they represent a unique phenotype. The fp-COA have smaller left heart structures (MV annulus, LV width, DTAA) compared to controls which is consistent with previous work [3, 4], but unlike the c-COA group, there was no compensatory increase in the size of the arterial duct. Doppler velocities and flow across the MV and TV are similar to c-COA and controls. The LV-CO was reduced in the fp-COA compared to normal and similar to the c-COA, again with preserved CCO. A key finding of our study is the identification of lower RVFW deformation in the fp-COA compared to c-COA and controls. RV deformation correlated with the size of the arterial duct raising the possibility that RVFW-GLS is impacted by the resistance imposed by a non-enlarged arterial duct (Table 5). The LV deformation was not different in the fp-COA compared to the c-COA which is consistent with other reports [9]. There was a non-significant trend (*p* = 0.053) towards lower LV-GLS in fp-COA cases compared to controls also suggesting a myocardial difference in the fp-COA. Whilst causation cannot be attributed from this cross-sectional study, it could be speculated that reduced LV-GLS, RV-GLS, and LV-CO in fp-COA are a maladaptive response to increased afterload in the form of anatomical restriction by the hypoplasia of the aortic arch and arterial duct. The multivariable regression analysis demonstrated an inverse relationship of LV-GLS to placental resistance in fp-COA, whilst there was no overall trend for differences in umbilical artery PI amongst the three groups studied, there is evidence of differences in the placentas of these fp-COA. A recent MRI study of placental oxygenation in fetuses with congenital heart disease, demonstrated lower markers of oxygenation of the placenta in fp-COA fetuses compared to c-COA [25]. The patterns identified appear unique to this heterogenous group and do not match other reported pathological phenotypes such as that reported in placental insufficiency or severe intrauterine growth restriction [26]. Further work is required to assess the contribution of the placenta to the phenotype of the fp-COA cohort.

### Comparison to Previous Studies

To our knowledge, there are no published studies comparing myocardial deformation, ventricular shape, and the interaction with afterload measures in fetuses with suspected COA. A number of studies have reported similar shape and hemodynamic findings in fetuses with c-COA: reduced LV sphericity, reduced LV-CO, increased RV-CO, increased MV inflow Doppler velocities, and reduced LV deformation, establishing that there is a difference in the function and geometry compared to normal fetuses [6–8]. However, the reason for these changes in fetuses with COA has not been investigated. Zeng et al. reported the association of myocardial deformation to aortic isthmus flow in fetuses with suspected COA and described a reduction in deformation with increasing flow resistance in the aortic isthmus, but did not describe whether this was isolated to the c-COA cases, fp-COA cases or the combined cohort [27]. The association between RV-SI and umbilical artery PI in the c-COA group is important and potentially, clinically relevant given the reported alterations in placental function in left-sided obstructive lesions [28]. In a large retrospective study, Devore et al. [9] used deformation amongst other cardiac parameters to assist in improving the differentiation between c-COA and fp-COA in the third trimester of pregnancy. Whilst the relationship of shape, size, and deformation was not studied, the results of the logistic regression analysis suggest an interaction of some measurements, but no assessment of the impact of afterload to the geometry and functional changes was made. The comparison is also limited as other structural and hemodynamic diagnoses such as mitral stenosis, partial anomalous pulmonary venous drainage, and Ebstein’s anomaly of the tricuspid valve were also included which may impact upon functional assessments.

The abnormal ventricular geometry and functional phenotype of fp-COA cases has been described in other echocardiographic studies [3, 9]. A recent study from our institution has also demonstrated this abnormal physiological phenotype of fp-COA using fetal cardiac MRI, demonstrating LV-CO, ascending aortic flow and aortic isthmus flow was reduced in fp-COA compared to normal and taking an intermediate position between normal fetuses and c-COA [5]. Only one study has described changes in ventricular length with strain imaging in fp-COA [9]; determinants of abnormal myocardial deformation in fp-COA cases have not been previously assessed.

In our cohort apart from 8/22 cases of a non-stenotic bicuspid aortic valve, there were no other abnormalities found postnatally in the fp-COA group, this differs from other groups which report a heterogenous outcome. [4, 29, 30]

## Limitations

This study assessed the deformation in relation to the shape, size, preload, and afterload at a single point in the third trimester of pregnancy in fetuses where abnormal ventricular and arterial appearances had been established in the second trimester following standard obstetric anomaly scans. The relationship between blood flow, cardiac function, and the size of cardiac structures is complex and may alter through gestation. Sequential study from the second trimester may provide further insights, but from a practical perspective recruitment of patients for research immediately after the initial mid-trimester scan where the diagnosis of coarctation was suspected, was not deemed appropriate. Strain assessment in the fetus remains a research tool, and there is no consensus regarding the optimal frame rate at which to acquire fetal studies. Accurate measurement of the magnitude of flow across the atrial septum is not possible by echocardiography, but it is acknowledged that it may be a marker of preload which can be altered in fetuses with COA. The pulmonary vascular bed was not studied due to the lack of validated echocardiographic measures but may influence RV deformation and shape.

## Conclusion

This study demonstrates that there are three different cardiovascular phenotypes of the c-COA, fp-COA, and normal fetuses. Differences in myocardial deformation of the right and left ventricles in fetuses with c-COA, fp-COA cases and normal fetuses, providing insight into the alterations in physiology that are present. The predictors of myocardial deformation varied and notably, myocardial deformation in the fp-COA cases related to the fetal circulation, particularly afterload.

## Supplementary Information

Below is the link to the electronic supplementary material.

Supplementary file1 (DOCX 11266 kb)

## Data Availability

No datasets were generated or analysed during the current study.
