# Supplementary material for: Myocardial Deformation and Its Relation to Ventricular Shape, Preload, and Afterload in Fetuses with Suspected Coarctation of the Aorta
Source: Pediatr Cardiol. 2025 May 8;47(3):1056–67. doi: 10.1007/s00246-025-03878-6 (PMC12901095; doi:10.1007/s00246-025-03878-6)
Supplement: Supplementary file 1 — Supplementary file1 (DOCX 11266 kb) [file 246_2025_3878_MOESM1_ESM.docx]

### Supplementary figure 1: Correlation between global longitudinal strain and other variables

***
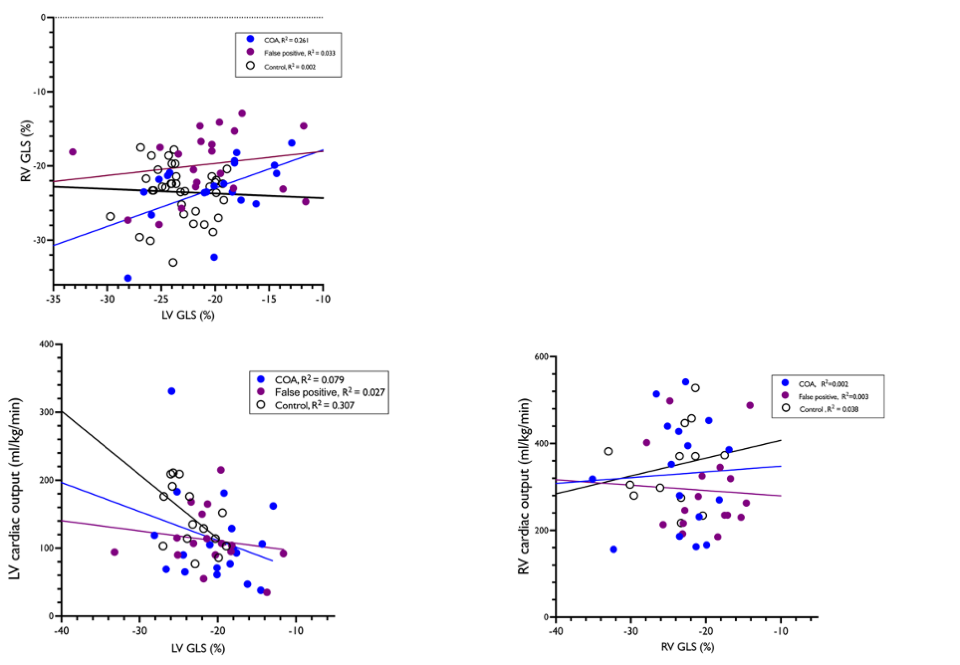
***

### Supplementary figure 2: Correlation between global longitudinal strain and variables impacting LV afterload

***
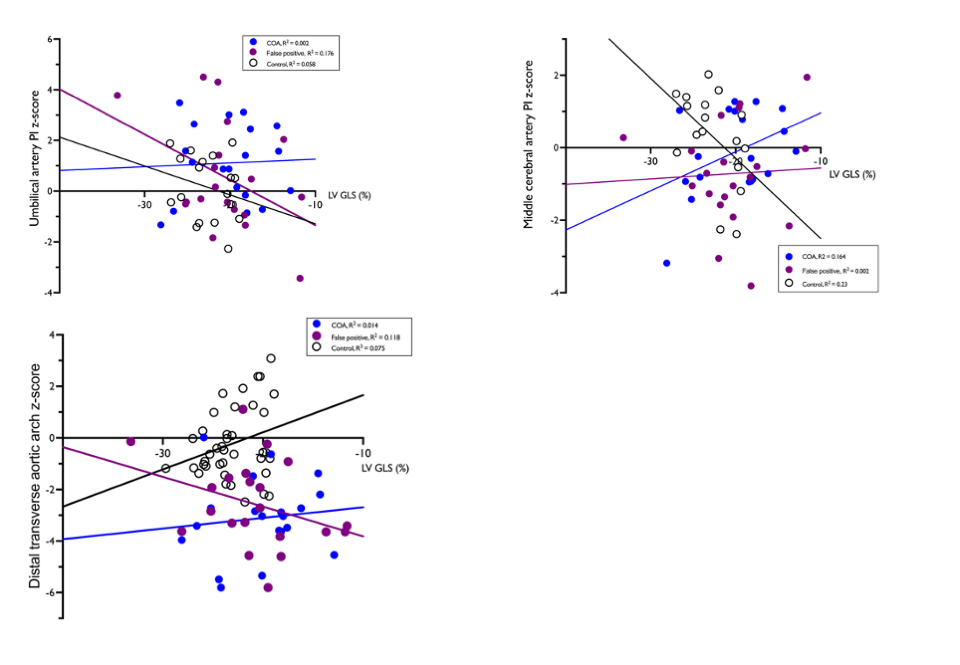
***

### Supplementary figure 3: Correlation between global longitudinal strain and ventricular sphericity

***
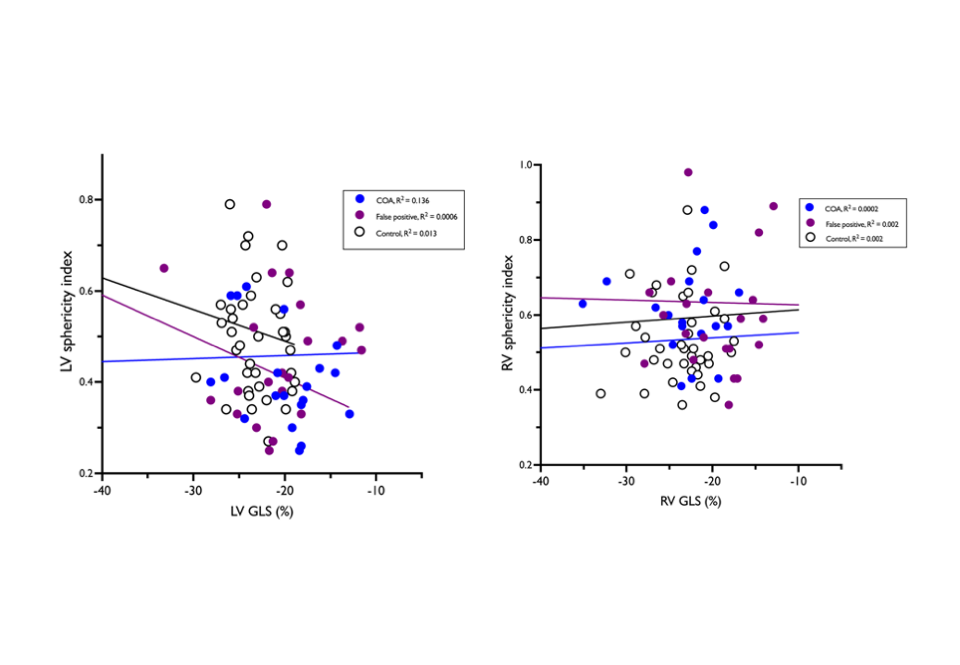
***

### Supplementary figure 4: Correlation between global longitudinal strain and variables influencing RV afterload

***
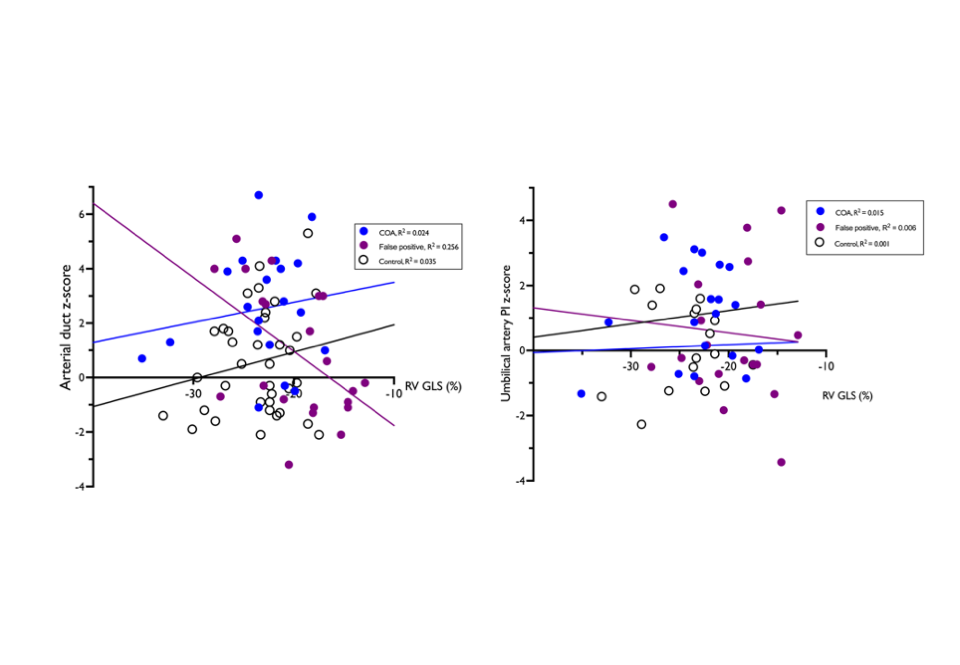
***

### Supplementary figure 5: Correlation between LV sphericity index and other measured variables

***
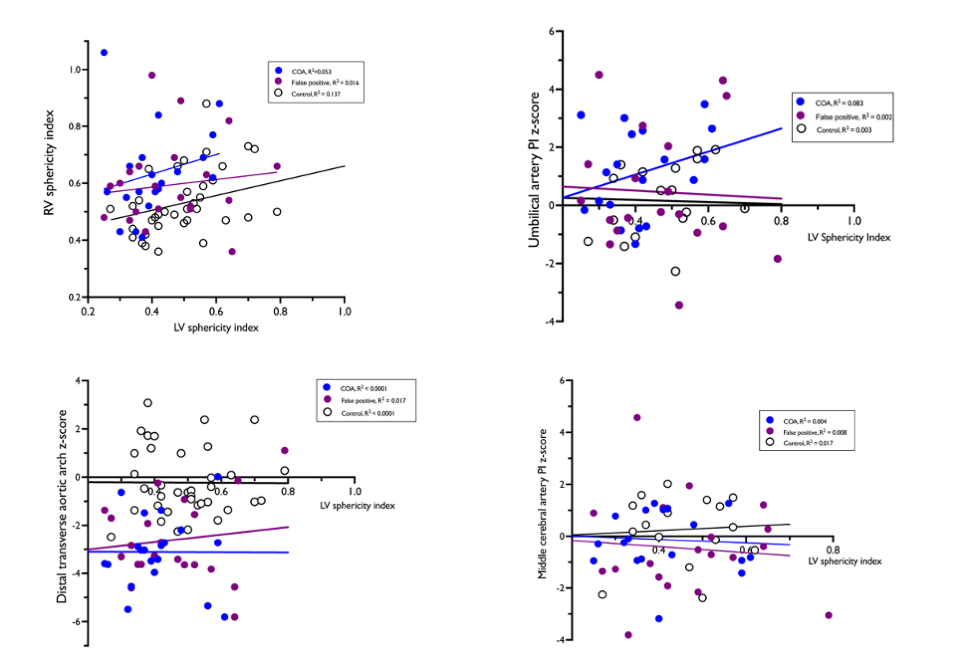
***

### Supplementary figure 6: Correlation between RV sphericity index and other measured variables

***
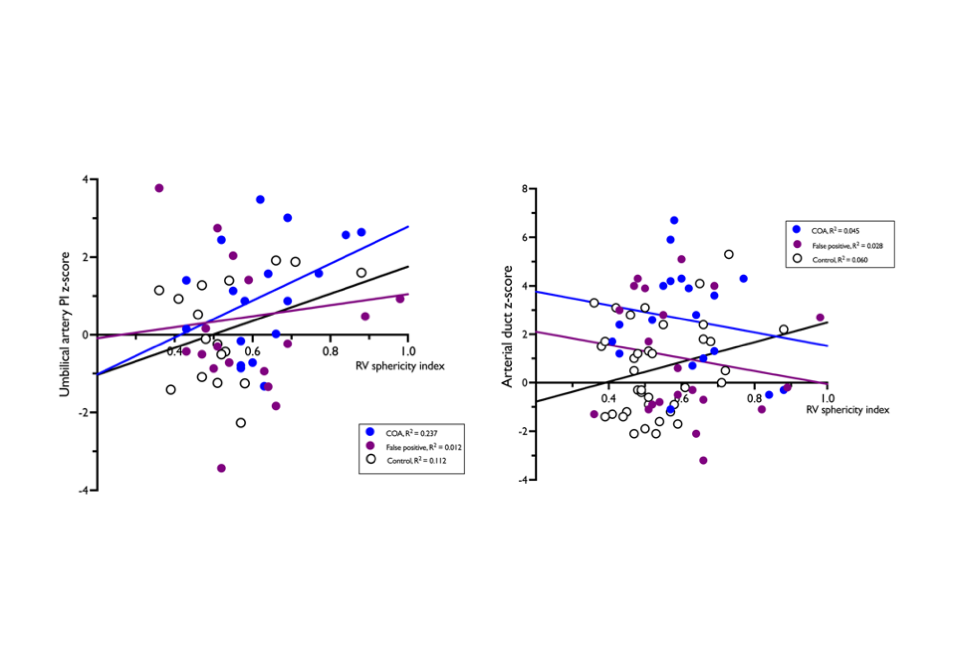
***
